# Excess free fructose, apple juice, high fructose corn syrup and childhood asthma risk – the National Children’s Study

**DOI:** 10.1186/s12937-020-00578-0

**Published:** 2020-06-23

**Authors:** Luanne R. DeChristopher, Katherine L. Tucker

**Affiliations:** 1 Independent Researcher, M.Sc. Biochemistry, Molecular Biology, NY Medical College, Valhalla, NY USA; 2 Department of Biomedical and Nutritional Sciences, University of Massachusetts Lowell, Lowell, MA USA

**Keywords:** Asthma, High fructose corn syrup, Excess free fructose, Apple juice, Juice, Fructositis, Fructose, Soda, Soft drinks, Fruit drinks, Advanced glycation end-products, AGE, FruAGE, Microbiome, RAGE, FODMAP

## Abstract

**Background:**

Recent research provides consistent evidence that the unexplained doubling of childhood asthma prevalence (1980–1995), its continued climb and 2013 plateau, may be associated with the proliferation of high-fructose-corn-syrup (HFCS) in the US food supply. The HFCS used in soft drinks has been shown to contain a higher fructose-to-glucose ratio than previously thought. This coincides with a preference shift from orange to apple juice among young children. Apple juice naturally contains a high (≥2:1) fructose-to-glucose ratio. Thus, children have received high excess-free-fructose doses, the fructose type associated with fructose malabsorption. Unabsorbed excess-free-fructose in the gut may react with dietary proteins to form immunogens that bind asthma mediating receptors, and/or alter the microbiota towards a profile linked to lung disorders. Studies with longitudinal childhood data are lacking. Therefore, we tested the hypothesis that excess-free-fructose intake is associated with childhood asthma risk.

**Methods:**

Cox regression models were used to analyze prospective early childhood data (12–30 months of age) from the National Children’s Study. Intake frequencies for soda/sports/fruit drinks, and 100% juices were used for analyses.

**Results:**

Greater consumption of 100% juice, soda/sports/fruit drinks, and any combination, was associated with ~two (*P* = 0.001), ~ 2.5 (*P* = 0.001), and ~ 3.5 times (*P* < 0.0001) higher asthma incidence.

**Conclusions:**

Given these results, prior research and case-study evidence, it is reasonable to suggest that the two-fold higher asthma risk associated with 100% juice consumption is due to apple juice’s high fructose-to-glucose ratio, and that the ~ 2.5/~ 3.5 times higher risk associated with soda/sports/fruit drinks intake is with the excess-free-fructose in HFCS.

## Background

The unprecedented doubling of childhood asthma prevalence from 1980 to 1995, its continued climb and 2013 plateau, mainly due to plateauing prevalence among non-Hispanic black children [1], has been described by the US Center for Disease Control (CDC) as mysterious [2]. African American, Puerto Rican and children from the poorest families, have been hardest hit by the epidemic [1–3]. One in 9 children has asthma [1–3]. Asthma sufferers have higher prevalence of chronic pro-inflammatory diseases including, but not limited to, inflammatory bowel disease [4], depression [5], and coronary heart disease [6]. Obesity has been ruled out as a cause, as a recent CDC study found that the unexplained rise in asthma prevalence was among normal weight children [3]. Their findings controverted a prior hypothesis, that increasing weights were responsible for the increased asthma incidence [3]. Air quality is an unlikely cause, as it has improved significantly due to > 40 years of clean air policies, since passage of the Clean Air Act (1970) [7], and this period coincides with significant reductions in tobacco smoking [8]. In a nationally representative sample, researchers found no evidence that early childhood exposure to air toxins increased asthma risk [9].

Recent case study [10] motivated epidemiological [11–16], biochemical [17–19], and other research [20–28] provides consistent evidence that the asthma epidemic may be associated with the ubiquitous presence of high fructose corn syrup (HFCS) [29, 30] in the US food supply. Results [10] of a rigorous food elimination diet [31], in a normal weight 2–3 year old, provided evidence that HFCS intake triggers gastro-intestinal distress, airway mucus hypersecretion that overwhelms the airways, chronic bronchitis, asthma and joint pain. There was no association with sucrose [10].

Excess-free-fructose is fructose which occurs when the fructose-to-glucose ratio exceeds 1:1, as in HFCS and apple juice. Most natural foods have a 1:1 fructose-to-glucose ratio. Apples/apple juice/pears/watermelons/and mangoes are among the exceptions [32]. The case study [10] led to elucidation of a mechanistic (fructositis) hypothesis [10] which proposes that fructose malabsorption underlies the HFCS/excess-free-fructose/asthma/inflammation link, which by extension includes 100% apple juice, as it contains a ≥ 2:1fructose-to-glucose ratio (National Nutrient Database (NDB) #09400) [32]. Unabsorbed excess-free-fructose in the gut reacts with partially digested dietary proteins to form immunogens via a reaction known as glycation. These immunogens trigger inflammation, gastro-intestinal, respiratory, and tissue distress. Research aimed at testing this hypothesis with nationally representative health data showed that modest (1–4/wk) and regular (≥5/day) excess-free-fructose intakes, gauged by intake of any combination of apple juice/fruit drinks/regular soda, were associated with four and five times higher likelihood of childhood asthma, relative to never/seldom consumers (*p* = 0.035/*p* = 0.005) [11]. Longitudinal research with adults was also significant [12]. In both studies, there was no association with orange juice. Apple and orange juice contain comparable amounts of total sugars and post-pasteurization antioxidants, per cup [32]. What distinguishes them is their fructose-to-glucose ratio. Unlike apple juice (≥2:1, NDB #09400), orange juice contains a ~ 1:1 fructose-to-glucose ratio (NDB #09209) [32].

Popular sodas have contained more fructose [33, 34] than the 55% that is generally-recognized-as-safe (GRAS) [35]. This practice may extend to foods. Intake of HFCS that is not GRAS is a problem for fructose malabsorbers [36–38]–a condition that appears to be man-made, as most unprocessed foods contain a ~ 1:1 fructose-to-glucose ratio [32]. Fructose malabsorption prevalence is higher in non-Hispanic blacks [39] which may explain the black/white childhood asthma disparity which inexplicably grew twofold from the 1980’s to 2010 [1, 2]. Children are sensitive at lower excess-free-fructose intakes than adults [36, 37], which may explain the doubling of childhood asthma prevalence (1980–1995) and its continued climb through 2013 [1–3]. Recent 100% juice reductions in the Special Supplemental Nutrition Program for Women, Infant, and Children [40] and food manufacturers’ displacement of HFCS with other sweeteners, may account for the recent plateauing prevalence among non-Hispanic black children [1].

### Study objectives

We aimed to epidemiologically test the hypothesis that increasing excess-free-fructose intake is associated with higher asthma risk in children. Survival analysis was conducted with data from the National Children’s Study (NCS) [41]. Beverage intake data were used for analyses, including intake frequency of soda/sports/fruit drinks, and 100% juices. Although the survey did not distinguish between 100% juice types, we anticipated that 100% juice intake, among children (ages ~ 12–30 months), would be associated with increased asthma risk, as apple juice is the most consumed juice by children < 5 years of age [42, 43], its consumption doubled from 1980 to 2010 [44], as measured by loss adjusted food availability data (LAFA), and apple juice is a main ingredient/sweetener in 100% juice blends marketed to children. Studies from the early 1990’s [43], and again in 2008 [42], documented the preference shift away from orange juice (1950’s) to apple juice among this age group. Data from the 2008 Feeding Infants and Toddlers Study, showed that more toddlers consumed apple juice (33.1%) than grape (6.2%) or citrus/citrus juice blends (16.6%) [42]. During the study period, HFCS was the main sweetener in US soda [30].

We used beverage intake data to test our hypothesis, as research aimed at measuring the fructose content in popular sweetened beverages found that the fructose-to-glucose ratio was on average higher (1.5:1) [33, 34] than the 1.2:1 that is GRAS [35]. In some popular brands this ratio was as high as 1.9:1 [33, 34]. Many varieties of fruit drinks are sweetened with HFCS and apple juice, and many varieties of sports drinks are sweetened with fructose. The fructose-to-glucose ratio in 100% apple juice (≥2:1, NDB No. 09400) [32] is higher than soda.

This is the first study that we know of, to epidemiologically test this hypothesis using prospective early childhood data.

## Methods

### Participants

The NCS was planned as a long-term prospective study of U.S. children and their parents. The pilot phase began in 2009. The study would have followed 100,000 children from before birth to age 21 [41]. However, the study was closed in December 2014, for reasons that have been described elsewhere [45]. When recruitment ended in 2013, the study had enrolled approximately 5000 children. By the 30 month exam there were 3011 participants.

We analyzed prospective data from two time periods, the twelve to eighteen-month exam, and the eighteen through 30-month exam. Asthma status was ascertained at the 18 and 24-month exams. Beverage intake patterns were obtained once, at the 30-month exam, via food frequency questionnaire. Questions included, “over the past 6 months how often did your child consume 100% juice/soda/sports/fruit drinks.” Although intake frequencies were obtained once, longitudinal research of children ages 2–5, has shown that intake patterns are established early and reflect consistent exposures to the same food groups, including 100% fruit juice and carbonated soft drinks, over time [46]. Further research has shown that the proportion of children who consume 100% juice and sweetened beverages has remained consistent between ages 1–3 [42, 47]. The NCS was authorized by the Children’s Health Act of 2000. Parents gave written consent [40].

There were 2118 participants with asthma and beverage intake data from the 18 through 30-month exams. This number was reduced to 2094 (100% juices) and 2097 (soda/sports/fruit drinks), due to missing weight status, vegetable/fruit intake, and birthdate data (model 1). Analysis models 2 and 3 had fewer participants due to missing smoke exposure/mother’s socioeconomic status (SES). A flow-chart showing exclusions is provided. See Supplemental Fig. 1**-** Flow Chart showing Exclusions and Sample Sizes.

### Beverage intake

We analyzed beverage intake data, individually and as any combination of both beverage types-soda/sports/fruit drinks and 100% fruit juices, herein referred to as ttlEFF. For individual analyses, intake frequencies were reduced from seven to three (≤2.5 times/wk., 3–7 times/wk., >once/day). To analyze ttlEFF intake frequencies, the following values were assigned, and then summed per respondent: zero for never; 0.286 and 0.714 for 1–3 and 4–6 times, during the past 7 days, respectively; 1 for once/day; 2 for 2 times/day; 3 for 3 times/day and 4 for 4 or more times/day. Sums were divided into quintiles. Strong and consistent relationships have been reported between frequency of food and food-group consumption obtained via food frequency questionnaires and probability of consumption on 24 h recalls [48]. Intakes derived via frequency questionnaires reflect usual intake patterns [48].

### Ascertainment of endpoints

Incident asthma was defined as self-reported-doctor-diagnosed asthma, as ascertained initially during the 18 month exam and again at the 24 month exam.

### Potential confounders

Potential confounders were selected based on existing research [11, 22, 25]. In the first analysis model, adjustments were made for sex, age, race/ethnicity, self-reported doctor diagnosed overweight status, and intake frequency of fruits/vegetables. The latter three were obtained during the 30 month exam. For analyses, fruit/vegetable intake frequencies, obtained via a food frequency questionnaire, were reduced from seven to three (≤ 2.5 times/wk., 3–7 times/wk., >once/day). Mother’s race/ethnicity was used if the child’s was missing. In the second and third models (*n* = 1833/1830)/(*n* = 1790/1791), we further adjusted for hours exposed to in-door smoke, a time varying covariate, fast food intake frequency (model 2), and mothers education status-a measure of SES-which was reduced from six to two levels (≤high school graduate; some/college graduate) (Model 3). We adjusted for fast food intake frequency, as we were interested in assessing asthma risk independent of fast food consumption. Although existing research in children is limited, some adult studies have found that a “Western style” diet characterized by high meat consumption may be associated with asthma [49]. Fast food intake frequencies were reduced from seven to four (zero times/wk., 1–3 times/wk., 4–6 times/wk., ≥1 time/day), asked as, during the past 7 days, how many times did your child eat a meal or snack from a fast food restaurant…include eating/carry out and delivery of meals. We used in-door smoke exposure from the 18 month exam, if the 24 month exam data was missing (*n* = 183).

### Statistical analysis

Multivariable-adjusted and bivariate Cox proportional hazards models, with time on study as the time scale, were used for analyses. There were no exclusions for history of asthma, as incident asthma was asked about for the first time during the eighteen month exam. After verifying proportional hazards assumptions, using the Schoenfeld and scaled Schoenfeld residuals for the models, as a whole and individually (*P* ≥ 0.05), we examined incident asthma over approximately 18 months of follow-up. Person-time was calculated from approximately 12 through 30 months of age, or incident asthma. Twelve months was used if age was not provided at the twelve month exam.

R version 3.4.3 and RStudio version 1.1.383 were used, and a two-tailed *p* value < 0.05 with a 95% confidence interval that did not include 1 was considered statistically significant.

## Results

### Baseline characteristics

A higher percentage of children consumed 100% juices (23.3%) > once/day, relative to soda/sports/fruit drinks (6.2%). A larger proportion (82.2%) of children consumed soda/sports/fruit drinks never or occasionally (≤2.5 times/wk) as compared with 100% juices (46.9%). However, almost one-fifth of children consumed soda/sports/fruit drinks approximately every other day (3–7 times/wk) or more (>once/day). Most children (91.7%) were not exposed to in-door smoke. However, 5.5% of children were exposed to 1 h of smoke/day, and nearly 3% were exposed ≥2 h/day. Very few (2.7%) children were overweight, as told by a doctor. Few children (9.1%) consumed fast food ≥4 times p/wk. Vegetable intake was likely below US dietary guidelines (1 cup for a 24 month old) for most children, as only 39.2% consumed vegetables >once/day. A higher percentage of children (60%) consumed fruit >once/day. Most mothers (69.8%) had some college or were college graduates (Table 1).

**Table 1:** Characteristics of Children, the National Children’s Study, 18–30 months^1^

n	2097
**Sex** (% female)	49.0
**Race/Ethnicity (%)**	
Non-Hispanic white	46.5
Hispanic	11.1
Non-Hispanic black	7.6
Other	34.8
**Hours exposed to in-door smoke /day at 18 months (%) (*n*** **= 1832/ 1835)**	
None	91.7
1	5.5
≥ 2	2.8
**Child is overweight, as told by a doctor/healthcare professional**	
(24–30 months, %Yes)	2.7
**Intake frequency ttlEFF**^**2**^**(%)**	
**(any combination Soda/sports/fruit drinks & 100% juice)**	
≤ 2.5 times /wk	30.9
3 times /wk	9.4
4–7 times /wk	21.9
> 1–2 times /day	18.7
> 2 times /day	19.0
**Intake frequency 100% juice (%) (*n*** **= 2096)**	
≤ 2.5 times /wk	46.9
3–7 times /wk	29.8
> once /day	23.3
**Intake frequency soda/sports/fruit drinks (%)**	
≤ 2.5 times /wk	82.2
3–7 times /wk	11.6
> once /day	6.2
**Intake frequency fruits (%)**	
≤ 2.5 times /wk	9.5
3–7 times /wk	30.5
> once /day	60.0
**Intake frequency vegetables (%)**	
≤ 2.5 times /wk	16.9
2–4 times /wk	43.8
≥ 5 times /wk	39.2
**Intake frequency fast food (%)**	
zero times /wk	36.0
1–3 times /wk	54.9
4–6 times /wk	02.3
≥ 1 time /day	06.8
**Mother’s Education (%) (*n*** **= 1790/ 1793)**	
≤ Highschool graduate	30.2
Some college or college graduate	69.8
**Mother’s Household Income (%) (*n*** **= 849/ 852)**	
≤ $49,999	53.0
≥ $50,000	47.0

1) Characteristics are based upon (parent) self-reported responses to the medical/ food intake frequency questionnaire. 1) ttlEFF means any combination of high fructose corn syrup sweetened soda/sports/fruit drinks, and/or 100% juice

Children who regularly (3–7 times/wk) or frequently (>once/day) consumed 100% fruit juice and/or soda/sports/fruit drinks were the lowest consumers of vegetables (≤2.5 times/wk). Regular/frequent ttlEFF consumption was not associated with being overweight. Children who consumed fast food ≥1 time /day (6.8%) were more likely to consume juice > 1/d than soda/sports/fruit drinks. Regular/frequent fruit consumers were less likely to consume (≤2.5 times/wk) soda/sports/fruit drinks than less frequent consumers. Children whose mothers were college graduates or completed some college, versus having less education, were less likely to consume soda/sports/fruit drinks (Table 2).

**Table 2:** Characteristics of Children by Beverage Intake Frequency, the National Children’s Study

ttlEFF^a^ -Any Combination of Soda/Sports/Fruit Drinks, and 100% Juices (***n*** = 2097						100% Juices (***n*** = 2094)			Soda/Sports/Fruit Drinks (***n*** = 2097)		
**Intake Frequency**	**≤2.5/wk**	**3/wk**	**4–7/wk**	**> 1–2 /d**	**> 2/d**	**≤2.5 /wk**	**3–7/wk**	**>once/d**	**≤2.5/wk**	**3–7/wk**	**>once/d**
**%**	30.9	09.4	21.9	18.7	19.0	46.9	29.8	23.3	82.2	11.6	06.2
**Sex (%)**											
Female	16.0	04.6	10.8	08.6	09.1	23.2	14.2	11.7	41.1	05.5	02.5
Male	15.0	04.8	11.1	10.2	10.0	23.7	15.6	11.6	41.2	06.1	03.7
**Race/ Ethnicity**^b^											
NHW	16.1	05.4	10.3	07.6	07.2	24.5	13.6	08.5	39.6	04.3	02.6
Hispanic	02.5	00.8	02.3	02.8	02.7	04.2	03.7	03.2	08.5	01.8	00.8
NHB	00.7	00.2	01.5	02.1	03.0	01.5	02.6	03.4	04.8	01.6	01.2
Other	10.0	03.5	06.8	06.6	07.7	15.8	10.1	08.8	28.3	03.9	02.6
**Hours Exposed to in-home smoke/day at ~ 18 months (%) (*n*** **= 1832/ 1835)**											
< 2	29.0	09.0	20.6	17.4	16.1	44.1	27.6	20.1	76.7	09.7	05.4
2	01.3	00.2	01.0	01.0	02.0	02.1	01.5	02.0	03.8	01.0	00.7
> 2	00.3	00.2	00.5	00.6	01.1	01.0	00.7	01.1	01.5	00.9	00.3
**Overweight at 24–30 months (%)**											
No	30.4	09.2	21.5	18.0	18.2	45.9	29.0	22.4	80.7	10.9	05.8
Yes	00.5	00.2	00.4	00.8	00.7	01.0	00.8	00.9	01.7	00.6	00.4
**Fruit Intake**											
≤ 2.5 /wk	03.2	01.0	01.5	01.7	02.2	04.9	02.4	02.2	07.4	13.3	00.9
3–7 /wk	07.7	03.2	08.0	06.2	05.4	13.5	10.7	06.3	24.3	04.4	01.8
> 1/d	20.1	05.2	12.3	11.0	11.3	28.6	16.7	14.7	50.7	05.8	03.5
**Vegetable Intake**											
**≤** 2.5 /wk	04.8	02.1	02.6	03.3	04.2	08.2	04.1	04.6	13.4	01.8	01.8
3–7 /wk	13.1	04.0	10.8	08.4	07.5	19.8	14.3	09.8	36.2	05.6	02.1
> 1/d	13.1	03.3	08.6	07.0	07.2	18.8	11.5	08.9	32.8	04.2	02.3
**Fast Food Intake**											
zero times /wk	15.2	02.4	08.5	05.5	04.4	19.0	09.4	07.4	32.3	02.8	01.0
1–3 times /wk	14.0	06.7	11.6	10.8	11.7	24.7	17.4	12.7	44.4	06.8	03.7
4–6 times /wk	00.3	00.2	00.6	00.6	00.6	00.9	00.8	00.6	01.5	00.5	00.3
≥ 1 time /day	01.3	00.1	01.3	01.7	02.2	02.0	02.2	02.5	04.1	01.5	01.1
**Mother’s Education Level (%)*n*** **= 1790/1793**											
≤ HS/graduate	05.6	02.7	05.2	06.7	09.9	11.1	08.6	10.4	20.9	05.3	04.0
≤ College/Grad	25.3	06.8	16.8	11.8	09.2	36.0	21.2	12.8	61.4	06.1	02.3

^a^ ttlEFF means any combination of high fructose corn syrup sweetened soda/sports/fruit drinks, and/or 100% juice. ^b^Mother’s race/ethnicity was used if child’s was missing (894)

More than once a day fruit intake, being female and having normal weight appeared protective against asthma (data not shown). Asthma was significantly more likely among Hispanics and non-Hispanic blacks across most analysis models, independent of sex, age, overweight, beverage/fruit/vegetable intakes, and exposure to in-door smoke.

### Relationship with asthma

Moderate to daily intake (3–7 times /wk) of 100% fruit juice and soda/sports/fruit drinks was significantly associated with ~ 1.5 and ~ 2 times higher asthma risk relative to never/occasional consumption (≤2.5 times/wk), respectively. Among >once /day consumers, asthma risks were ~ 2 and ~ 3 times that of never/occasional consumers, respectively. The number of asthma cases/1000/year more than doubled (22 to 48) with increasing 100% juice consumption, and nearly tripled (31–84) with increasing soda/sports/fruit drinks consumption. Table 3.

**Table 3:** Childhood Asthma Risk according to Beverage Consumption, The National Children’s Study

***Incident Asthma***^a^	Cox Proportional Hazards Model			1 IR per 1000	Persontime years	Cases /1000 /year	Cox Proportional Hazards Model 2			Cox Proportional Hazards Model 3		
	Hazard Ratio	95% CI	***p***-value				Hazard Ratio	95% CI	***p***-value	Hazard Ratio	95% CI	***p***-value
	**HR** –adjusted for sex, age, overweight, race/ethnicity, fruit/ vegetable intake						**HR** – further adjusted for hours exposed to in-door smoke, fast food intake			HR – further adjusted for mother’s education level		
**ttlEFF**^b^**–any combination (Soda /Sports/Fruit drinks & 100% Juices)**	*n* = 2097, # of observations = 4193						*n* = 1833, # of observations = 3665			*n* = 1791, # of observations = 3581		
≤ 2.5 /wk	Reference -------			20	1140	18	Reference -------			Reference -------		
3 /wk	1.44	0.71–3.49	0.367	9	361	25	1.86	0.82–4.23	0.137	1.36	0.52–3.02	0.501
4–7 /wk	1.95	1.15–3.63	0.022*	28	799	35	2.29	1.25–4.22	0.008**	2.24	1.16–3.92	0.010*
> 1 & 2 /day	2.81	1.69–5.19	0.0003**	34	663	51	3.01	1.65–5.52	0.0003*	2.84	1.48–4.95	0.0008**
> 2 /day	3.59	2.19–6.60	0.000004***	40	636	63	3.62	1.98–6.62	0.00003***	3.24	1.69–5.67	0.0002***
**100% Juices**^**c**^	*n* = 2094, #of observations = 4187						*n* = 1830, # of observations = 3659			*n* = 1790, # of observations = 3575		
≤ 2.5 /wk	Reference -------			43	1967	22	Reference -------			Reference --------		
3–7 /wk	1.44	0.94–2.21	0.097∞	41	1250	33	1.56	1.00–2.44	0.049*	1.63	1.04–2.56	0.033*
> once /day	2.19	1.44–3.32	0.0002**	47	974	48	1.87	1.18–2.95	0.008**	1.82	1.14–2.92	0.013*
**Soda/Sports/Fruit drinks**												
	*n* = 2097, # of observations = 4193						*n* = 1833, # of observations = 3665			*n* = 1791, # of observations = 3581		
≤ 2.5 /wk	Reference -------			95	3051	31	Reference -------			Reference -------		
3–7 /wk	1.99	1.20–3.30	0.008**	20	357	56	2.08	1.20–3.60	0.009*	2.03	1.17–3.53	0.012*
> once /day	2.90	1.67–5.05	0.0002**	16	190	84	2.83	1.54–5.22	0.0008**	2.57	1.38–4.79	0.003*

Cox regression hazard ratios (HR), their 95% confidence intervals and p values are shown. Significant associations are denoted with asterisks. Multiple asterisks denote highly significant associations. Associations that approached significance are denoted by the ∞ symbol. ^a^ Asthma refers to the first incidence of asthma as self-reported during the 18 and 24 month exams. ^b^ ttlEFF refers to intake frequency of any combination of high fructose corn syrup sweetened soft drinks/sports/fruit drinks and 100% juice. ^**c**^ Juice types were not distinguished. The most consumed juice by children < 5 years of age is apple juice, and apple juice is widely used as a main ingredient/sweetener in 100% juice blends marketed to children [42, 43]. Per capita apple juice consumption doubled from 1980 to 2010 [44]. During the study period, HFCS was the main sweetener in US soda [30]. Many varieties of fruit drinks are sweetened with HFCS and apple juice. The fructose to glucose ratio in apple juice is ≥2:1.^[32]^In HFCS sweetened soft drinks the ratio has been measured as 1.5:1 for HFCS with 60% fructose [34] and 1.9:1 for HFCS with 65% fructose [33] – higher than the 1.2:1 ratio that is generally recognized as safe [35]^.^

Results with any combination of 100% juice and soda/sports/fruit drinks (ttlEFF) were highly significant across all analysis models (Table 3). Increasing consumption of any combination of ttlEFF was significantly associated with increasing asthma risk, from 95% higher among children who consumed ttlEFF 4–7 times/wk. (HR = 1.95/*P* = 0.022), to 181% higher among > 1 to 2 times/day consumers (HR = 2.81/*P* = 0.0003), to 259% higher among > 2 times/day consumers (HR = 3.59/*P* < 0.00001), relative to never/occasional consumers, adjusted for sex, age, race/ethnicity, overweight and fruit/vegetable intake. Results remained fairly consistent after further adjustment for in-door smoke exposure, fast food intake, and mother’s education level. The number of asthma cases/1000/year more than tripled (18–63) with greater ttlEFF consumption. Table 3. Fast food consumption *was not* associated with asthma risk across any of the analysis models (data not shown).

Unadjusted associations are provided in Table 4.

**Table 4:** Unadjusted Risk of Incident Asthma according to Beverage Consumption, The National Children’s Study

***Incident Asthma***^a^	Cox Proportional Hazards		
	Hazard Ratio	95% CI	***p***-value
**ttlEFF**^**b**^**(any combination of soda/sports/fruit drinks and 100% juice)**	*n* = 2118, # of obs = 4236		
≤ 2.5 /wk	Reference -------		
3 /wk	1.43	0.65–3.14	0.375
4–7 /wk	2.04	1.15–3.63	0.015*
> 1 & 2 /day	3.08	1.77–5.36	0.0000***
> 2 /day	3.89	2.27–6.67	0.000000***
**100% Juice**^**c**^	*n* = 2114, # of obs = 4228		
≤ 2.5 /wk	Reference -------		
3–7 /wk	1.52	0.99–2.32	0.057∞
> once /day	2.33	1.54–3.53	0.00000***
**Soda/sports/fruit drinks**	*n* = 2118, # of obs = 4236		
≤ 2.5 /wk	Reference -------		
3–7 /wk	2.22	1.35–3.67	0.002**
> once /day	3.33	1.92–5.75	0.0000***

Cox regression hazard ratios (HR), their 95% confidence intervals and *p* values are shown. Significant associations are denoted with asterisks. Multiple asterisks denote highly significant associations. Associations that approached significance are denoted by the ∞ symbol. ^a^ Asthma refers to the first incidence of asthma as self-reported during the 18- and 24-month exams. ^**b**^ ttlEFF refers to intake frequency of any combination of high fructose corn syrup sweetened soft drinks/sports/fruit drinks and 100% juice. ^**c**^ Juice types were not distinguished. The most consumed juice by children < 5 years of age is apple juice, and apple juice is widely used as a main ingredient/sweetener in 100% juice blends marketed to children [42, 43]. Per capita apple juice consumption doubled from 1980 to 2010 [44]. During the study period, HFCS was the main sweetener in US soda [30]. Many varieties of fruit drinks are sweetened with HFCS and apple juice. The fructose to glucose ratio in apple juice is ≥2:1.^[32]^In HFCS sweetened soft drinks the ratio has been measured as 1.5:1 for HFCS with 60% fructose [34] and 1.9:1 for HFCS with 65% fructose [33] – higher than the 1.2:1 ratio that is generally recognized as safe [35]

## Discussion

The most consumed juice by children < 5 years of age is apple juice [42, 43], and its consumption, as measured by US Department of Agriculture (USDA) loss-adjusted-food-availability data doubled from 1980 to 2010 [44]. Given these facts, and prior research [11–15, 22, 24], it is reasonable to suggest that the highly significant association between *daily* 100% juice consumption and the near doubling of asthma risk, independent of sex, race/ethnicity, fruit/vegetable intake (barometers of diet quality), fast food consumption, exposure to in-door smoke, overweight and SES, is with 100% apple juice–due to its high fructose to glucose ratio (≥2:1). Less frequent intake (3–7 times/wk) was associated with > 50% higher risk than never/occasional consumption. These findings are consistent with our cross-sectional analyses, wherein children who consumed apple juice (but not orange juice), 1–4 times/wk. and ~ daily, were nearly three and ~ 2.5 times as likely to have asthma as never/seldom consumers, respectively, independent of potential confounders [11].

Results of another study (Project Viva) are consistent with these results. Childhood intake of juice, excluding orange juice, was associated with asthma [22]. A subsequent re-analysis of Project Viva data bundled orange juice with other 100% juices [23]. Authors concluded that the asthma association was with all forms of fructose. However, this interpretation is inconsistent with their initial analysis results, [22] with our present and prior analyses of children [11], with the case study that motivated this research [10], and with adult survival analysis with 17 years of follow-up data [12] . In the latter study, moderate (2–4 times/wk) apple juice intake was associated with > 1.5 times higher asthma risk, versus never/seldom consumption, but orange juice–a 100% juice with a ~ 1:1 fructose to glucose ratio—was not [12]. Orange juice contains a similar amount of total sugars (20.7 g), and total fructose (11 g) [32], and has a similar glycemic load [50] (15 glycemic units) as apple juice (24 g)/(15.7 g)/(12 glycemic units) [32], per 250 ml (8 oz cup). However, they differ significantly in their excess-free-fructose content–the type of sugar associated with fructose malabsorption-which is 7.4 g in apple juice versus 0.4 g in orange juice, per 250 ml [32].

These results, combined with a CDC report, which found that the asthma epidemic that began in 1980 was mainly among *normal weight* children [3], provide further evidence that adiposity-mediated effects of all forms of fructose, as described by Wright et.al [23], do not explain the childhood asthma epidemic. Rather, the association appears to be with the high fructose- to-glucose ratio. This repeatability is consistent with pediatric fructose malabsorption (FM) research, wherein FM was highly associated with apple juice intake [37]. This 1995 study of six and eighteen-month old children showed that FM occurred more frequently (*p* < 0.001) after consumption of apple juice (54%) than after grape juice (1:1 fructose to glucose ratio) (19%) [37]. Children were given juice amounts to equate to 1 g fructose/kg body weight, about 2/3 of a cup (~ 160 ml) for an eighteen month old weighing ~ 10 kg (22 lbs.). Notably, 2/3 of a cup of apple juice contains about 6 g of excess-free-fructose versus 1.4 g in grape juice [32]. Six grams is lower than ~ 10 g to 25 g that is associated with adult FM [38]. Children are more sensitive at lower excess-free-fructose intakes than adults [36, 37].

Research has shown that Hispanic and non-Hispanic black children were more likely to consume fruit/soft drinks [51, 52], and 100% juice [52], than were non-Hispanic white children; and that poor, relative to non-poor children, were more likely to consume fruit drinks [51, 52]. This propensity may explain the race/ethnicity/SES disparities in childhood asthma prevalence/severity that began coincident with the US shift from sucrose to HFCS [1–3], as FM prevalence is higher among non-Hispanic blacks than whites [39]. Researchers have hypothesized that the soda/asthma link may be due to the preservatives in soda [25]. However, a literature review showed no strong evidence linking preservatives in soda with asthma [15]. Diet-soda contains the same preservatives, and diet-soda has not been associated with asthma. Furthermore, anionic ligands, including phosphates from soda and pancreatic bicarbonate injected into the duodenum after a meal, are potent catalysts of glycation. Overall, the scientific evidence points to the HFCS, rather than the preservatives [15].

Beverage industry sponsored research found that the method used to measure the fructose-to-glucose ratio in the HFCS in popular sodas was not sufficiently sensitive to detect the presence of 5–8% of maltose/small chain glucose oligomers [53]. However, this does not appear relevant, in the context of fructose malabsorption, as there is no evidence that we know of, which shows improvements in excess-free-fructose absorption with co-ingestion of maltose/short chain glucose oligomers. Experimentation with adding glucose to meals containing excess-free-fructose to enhance fructose absorption is largely based upon studies with pure simple sugar solutions (fructose/glucose monosaccharides) in healthy subjects [54]. Importantly, recent research [54] found that co-ingestion of glucose *did not* appear to improve fructose absorption or symptoms when applied to whole food containing fructose in excess of glucose. Breath hydrogen responses were not significantly attenuated in patients or in healthy controls and food induced symptoms were not altered. Results provide evidence that glucose co-ingestion is an *ineffective* strategy to reduce excess-free-fructose related abdominal symptoms [53]. Case study evidence [10] is consistent with their findings, as HFCS sweetened cold cereals and breads-foods that contain maltose [55]-triggered airway mucus hypersecretion/respiratory distress including asthma/chronic bronchitis [10]. These observations are consistent with another recent (2018) study. Researchers sought to examine the impact of different fructose-containing sweeteners (sucrose, HFCS 55/45%, fructose) on the intestinal, hepatic, and oral bioavailability of fructose via animal models *not modified to mimic malabsorption*. After normalizing for fructose dose, researchers found that co-ingestion of glucose did not enhance fructose absorption, rather, it decreased fructose metabolism in the liver [56].

There is consistent evidence, across age groups, that adherence to a Mediterranean diet, as characterized by high fruit and vegetable intake is protective against asthma [49]. Herein, we found that asthma risk among toddlers was significantly associated with moderate (3 times per week) and daily intake of 100% juice, and soda, sports/fruit drinks, and any combination, independent of fruit/ vegetable intake and weight status.

This study and prior research results [11–28] are consistent with the “fructositis” hypothesis^[10]^-that unabsorbed unpaired fructose in the gut of fructose malabsorbers, forms immunogens which activate receptors (RAGE) that are most highly concentrated in the lungs [57], and are associated with goblet cell hyperplasia with mucus hypersecretion, and bronchial/vascular/interstitial eosinophilia [58]. At physiological pH, fructose is in open chain form 400 times more than glucose [59]. This underlies its higher relative reactivity. When it remains unabsorbed, its reactivity fosters gut formation of immunogens via interaction with dietary proteins at varying stages of digestion [10]. This is consistent with evidence that these immunogens, *N*-ε-carboxymethyl-lysine (CML) and *N*-ε-carboxyethyl-lysine, bind asthma mediating receptors (RAGE) [58] independent of amino acid specificity [60]. There is no FM after consumption of 1:1 fructose to glucose ratios [36–38], except to some degree in infants and toddlers [37]. Hence, consequences of FM extend beyond gas/abdominal pain/decreased tryptophan absorption.

Biochemical research has confirmed the plausibility of intestinal glycation/immunogen formation. Pro-inflammatory intermediates [17] and CML-a well-studied AGE-formed in intestinal conditions with fructose, but not glucose, within the time-frame of digestion [18, 19]. In other research, John’s Hopkins investigators [61] failed to find a hypothesized correlation between elevated concentration of serum/ urinary CML and intake of foods thought to be high in dietary AGEs, including grilled/broiled meats and French fries (Energetics Study). Rather, a review [20] showed that CML associations were with intake of foods that were significant sources of HFCS [29, 30] including cold breakfast cereals, whole grains/breads, and sweets/desserts. Intake of fruit drinks approached significance. Inclusion of *sucrose*-sweetened beverages (coffees and teas) may have prevented a true assessment of CML associations with HFCS sweetened soda [20, 61]. In a similar study, CML concentration was unexpectedly higher in vegetarians, who consumed more apples/apple juice, than in omnivores [62]. Research that disambiguates the protein sources of serum/urinary CML via specialized mass spectrometry methods is needed.

Air quality and smoking have been suggested as possible causal factors, but are implausible explanations for the childhood asthma epidemic/black/white asthma disparity [1–3]. Air quality has improved significantly since the 1970’s [7, 8] and smoking rates are down comparably/uniformly across races [63]. Gut immunogen formation may explain the disparity, as non-Hispanic blacks have higher fructose malabsorption prevalence [39] than whites. Increases in childhood asthma began in 1980 [1]–a period that coincides with higher HFCS intakes (average per capita of approximately 1/3 of a lb./wk) [44], as reported before subjective, retroactively applied consumer level loss (LAFA) increases [64]. By 1999, intakes rose to more than a lb./wk. (~ 80 g/d) [44].

In 65 g of HFCS (~ 1 lb./wk) that exceeds GRAS levels, there are 13–19.4 g of excess-free-fructose, depending on the fructose percentage (60% [34] or 65% [33]), versus 6.4 g in HFCS that is GRAS [35]. Therefore, a 2 year old who consumes 1000 kcal/day may consume 6.5–9.7 g of excess-free-fructose from HFCS. This dosage exceeds levels (~ 5.5 g of excess-free-fructose from 2/3 of a cup of apple juice) associated with > 50% fructose malabsorption in 18 month olds [37]. The 2015–2020 US Dietary Guidelines recommend that at least half of fruit intake (1 cup for a 2 year old) should come from whole fruit [65]. Therefore, a 24 month old who consumes 1/2 cup of 100% apple juice/day, which contains ~ 4 g of excess free fructose [32], is following a healthy eating pattern, as currently defined [65]. For a 2 year old who consumes 1000 kcal/day, this dosage exceeds FM range when the daily excess-free-fructose intake from HFCS is factored in, irrespective of whether the HFCS is above GRAS (6.5–9.7 g) or at the GRAS (3.2 g) level. Recent replacement of HFCS with crystalline-fructose, and agave syrup (≥60 fructose) [32] may be exacerbating conditions for fructose malabsorbers.

Another mechanism that may link excess-free-fructose intake with asthma involves the microbiome. Research shows that increases in luminal fructose concentration affect gut bacterial load and composition, and reduce gut bacterial diversity [66]. Changes in the composition of the intestinal and airway microbiota have been associated with chronic lung disorders and respiratory infections [4]. The intestine can play a critical role in directing immune responses outside the local environment, including the lung [4]. Researchers have hypothesized that there is systemic dissemination of metabolites that originate in the intestinal microbiota and are involved the gut lung axis [4]. It is possible that a fructose induced shift in microbiome composition results in the proliferation of metabolites that bind asthma mediating receptors, which promote airway mucus hypersecretion, hyper-reactivity and chronic lung disorders. Antibiotic treatment of recurrent airway mucus hypersecretion/asthma which progresses to airway infection [67] may also have a disrupting effect on the microbiome, with lifelong consequences in the composition and function of the gut [68] and lung [4] ecosystems. Another possible mechanism may involve fructose induced metabolic dysregulation via altered dopamine signaling [69], which has been shown to exert effects on the airways [70].

Existing research has improved our understanding of the mechanisms by which fructose intake contributes to cardio-metabolic disease [71–73]. However, evidence to explain why some individuals are malabsorbers and others are not is lacking. Longstanding research shows that the intestine’s capacity to absorb free fructose is saturable and ranges widely from ~ 5 g to > 50 g in healthy adults [72]. The variability in the expression or function of the predominant fructose transporter, GLUT5, or its regulatory factors may contribute to the variability in fructose absorption in humans [72]. This apparently normal variability likely reflects the fact that most natural foods contain a near 1:1 fructose-to-glucose ratio, with minimal amounts of excess-free-fructose (EFF). Therefore, a person at the lower end of the range may not encounter problems unless they eat a large amount of apples (~ 4.3 g EFF/medium-sized apple) [32], pears (~ 5.9 g EFF/medium-sized pear) [32], mangoes (~ 4.4 g EFF/mango) [32], and watermelon (~ 2.8 g EFF (1 diced 8-oz cup) [32]. This should be considered in the context of food safety guidelines that govern high fructose sweeteners and apple juice/ apple juice blends.

This study has limitations. First, analyses were limited to beverage sources of HFCS which prevented evaluation of the true asthma risk associated with excess-free-fructose intake from HFCS, as food source data was not available. Second, due to misclassification bias, some children may have their symptoms diagnosed as something other than asthma which could have introduced a margin of error into our results. However, results are consistent with prior research [11, 12, 21–27], including one study wherein excess-free-fructose intake was associated with allergic symptoms and allergy sensitization [24]. Another limitation is that dietary patterns were obtained once at the 30 month exam, asked as “over the past six months how often did you consume..?” Therefore, the data reflect intake patterns at 24–30 months, and may not as closely reflect 12–18 month intake. However, existing research shows that intake prevalence at 12 months [42, 48] and usual intake patterns [46] of 100% juice and soda/fruit drinks persist through ages two and 3 years. Furthermore, results are consistent with case study evidence [10], and prior studies,^[11–28^] including research wherein soda intake was associated with doctor diagnosed active asthma among hospital admitted patients [27]. Another limitation is that 100% juice types were not distinguished, which limited our ability to measure the true risk associated with apple vs. other juices. However, there is consistent evidence that 100% apple juice is the most consumed juice in children < 5 years of age [42, 43]. Furthermore, in prior studies [11, 12, 22], apple juice intake was associated with asthma, not orange juice. The NCS Vanguard data is not nationally representative, thus, the conclusions may not be fully generalizable. However, these results are consistent with prior research based upon nationally representative health survey data of children [11].

## Conclusion

This is the first study of excess-free-fructose containing beverage intake and asthma with longitudinal children’s data. Results are consistent with existing research/case study evidence. The 100% juice/soda/sports/fruit drinks/asthma link in toddlers appears to be with the excess-free-fructose in apple juice/HFCS. Recommendations to limit consumption are inadequate, as associations are evident even at moderate intakes, and results don’t reflect the added risk from the HFCS/crystalline fructose/agave syrup in food. Even with education, navigating the food supply is difficult when these sweeteners are ubiquitous in the food supply. Without adequate labels and warnings, these sweeteners will continue to be a public health hazard.

## Supplementary information

**Additional file 1: Figure S1.** Flow Chart showing Exclusions and Sample Sizes.

## Data Availability

National Children’s Study data will be transferred to the National Institute of Child Health and Human Development (NICHD) Data and Specimen Hub (DASH) in 2020 after removal of HIPAA identifiers.

## Abbreviations

AGE: Advanced Glycation End-products
CDC: United States Centers for Disease Control
CI: Confidence Interval
CML: *N*-ε-carboxymethyl-lysine
DASH: Data and Specimen Hub
dAGE: Dietary Advanced Glycation End-products
EFF: Excess Free Fructose
enFruAGE: Extracellular newly-identified Fructose associated Advanced Glycation End-products
FFQ: Food Frequency Questionnaire
GRAS: Generally Recognized as Safe
HFCS: High Fructose Corn Syrup
HR: Hazard Ratio
NCS: National Children’s Study
NDB: National Nutrient Database
NHANES: (US) National Health and Nutrition Examination Survey
NIAID: (US) National Institute on Allergy and Infectious Diseases
NICHD: (US) National Institute of Child Health and Human Development
OR: Odds Ratio
RAGE: Receptors of Advanced Glycation End-products
SES: Socio-economic Status
SSB: Sugar Sweetened Beverages
ttlEFF: Any combination of beverages high in excess free fructose (fructose to glucose ratios that exceed 1:1) including high fructose corn syrup sweetened soda/sports/fruit drinks, and apple juice
US: United States
WIC: US Special Supplemental Nutrition Program for Women, Infants and Children
