# Supplementary material for: Excess free fructose, apple juice, high fructose corn syrup and childhood asthma risk – the National Children’s Study
Source: Nutr J. 2020 Jun 23;19:60. doi: 10.1186/s12937-020-00578-0 (PMC7313206; doi:10.1186/s12937-020-00578-0)
Supplement: Supplementary file 1 — Additional file 1: Figure S1. Flow Chart showing Exclusions and Sample Sizes. [file 12937_2020_578_MOESM1_ESM.docx]

**Figure 1. Flow Chart showing Exclusions and Sample Sizes**

**Initial sample:** Initial enrollment in the National Children’s Study - 5000 children

**Exclusions:**

Children lost to follow-up by the 30 month exam = 1989

Children with missing soda/sports/fruit drink and 100% juice intake data=73/71

Children with missing asthma status at the 18/24 month exam data=820

Subtotal n=2118

**Samples for asthma analysis by analysis model**:

n=2099/2096 participants for model 1 analysis of soda/sports/fruit drinks/ 100% juice (missing overweight status, and fruit/ vegetable intakes, n=19/22)

n=1835/1832 participants for model 2 analysis of soda/sports/fruit drinks/ 100% juice (missing hours exposed to in-door smoke, n=264/267)

n=1793/1790 participants for model 3 analysis of soda/sports/fruit drinks/ 100% juice (missing mother’s education level, n=42/45)
